# Machine Learning-Assisted Plasma Metabolomics Identifies a Five-Metabolite Panel for Colorectal Cancer Detection

**DOI:** 10.1021/acsomega.6c00857

**Published:** 2026-04-16

**Authors:** Jun-Kai Wong, Chung-Hsien Lin, Hsin-Yi Wu, Yen-Ping Lin, Chiau-Jun Chu, Pang-Hung Hsu, Chung-Fa Chang

**Affiliations:** † Department of Medical Laboratory Science and Biotechnology, College of Medicine, 34912 National Cheng Kung University, Tainan 70101, Taiwan; ‡ Instrumentation Center, 33561 National Taiwan University, Taipei 106, Taiwan; § 210849 Public Health Bureau, Tainan City Government, Tainan 701, Taiwan; ∥ Department of Bioscience and Biotechnology, 34880 National Taiwan Ocean University, Keelung 202, Taiwan; ⊥ Institute of Biochemistry and Molecular Biology, National Yang Ming Chiao Tung University, Taipei 112, Taiwan; # Center of Excellence for the Oceans, National Taiwan Ocean University, Keelung 202, Taiwan; ¶ Institute of Basic Medical Science, College of Medicine, National Cheng Kung University, Tainan 70101, Taiwan; ∇ University Center for Bioscience and Biotechnology, National Cheng Kung University, Tainan 70101, Taiwan; ○ Department of Pathology, National Cheng Kung University Hospital, College of Medicine, National Cheng Kung University, Tainan 70101, Taiwan

## Abstract

Colorectal cancer (CRC) remains a leading cause of cancer-related mortality worldwide, and improved noninvasive strategies for early detection are urgently needed to complement current screening approaches. We applied an integrated plasma metabolomics workflow that combines untargeted and targeted liquid chromatography–mass spectrometry with machine-learning-assisted feature prioritization. Plasma samples from a discovery cohort of 172 CRC patients and 115 healthy controls were analyzed, followed by validation in an independent cohort of 47 CRC patients and 47 healthy controls. From 146,880 spectral features, random forest analysis was used to robustly rank features, followed by ROC-based prioritization and metabolite annotation. Diagnostic performance was evaluated by using a logistic regression model. Phenotypic assays were conducted to assess the biological activity of selected metabolites in CRC cell models. Five metabolites, *N*-methylcytisine, 2-piperidone, theophylline, dl-norleucine, and linolenic acid, were consistently reduced in CRC patient plasma across both cohorts. The integrated five-metabolite panel achieved an area under the ROC curve of 0.968 in the validation cohort with a 97.9% sensitivity, an 89.4% specificity, and a 93.7% accuracy. Stratified analyses demonstrated robustness across disease stages and age groups. In vitro assays showed modulation of CRC cell migration and invasion under noncytotoxic conditions. This five-metabolite plasma signature reflects CRC-associated systemic metabolic alterations and demonstrates a strong discriminatory performance. The panel may complement existing screening modalities by contributing to CRC risk stratification and early detection.

## Graphical abstract

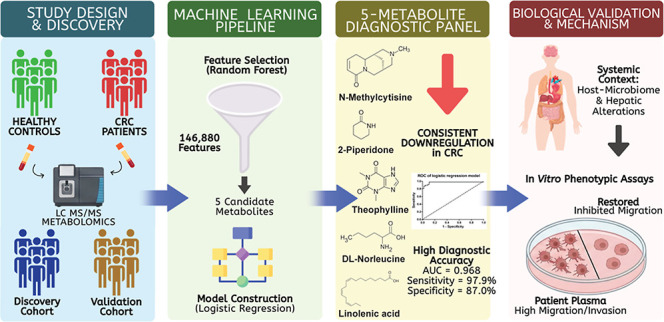

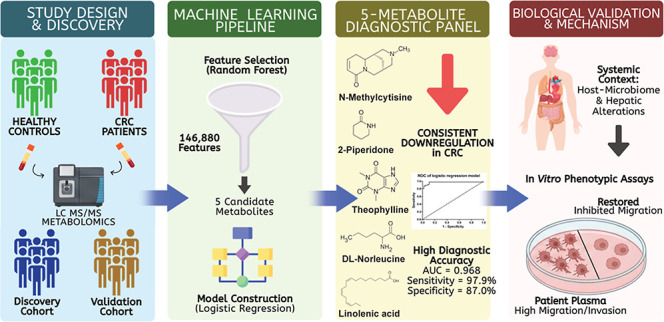

## 1 Introduction

Colorectal cancer (CRC) is the third most common cancer and the second-leading cause of cancer-related mortality worldwide. According to recent global estimates, more than 1.9 million new cases are diagnosed annually, with mortality exceeding 900,000.^1^ CRC typically progresses from adenomatous polyps to invasive carcinomas over 5–10 years,^2–5^ providing a critical window for early detection and intervention that substantially improves survival.

Current diagnostic approaches for CRC have notable limitations. Colonoscopy remains the gold standard because of its high sensitivity and ability to remove precancerous lesions;^6–10^ however, it is invasive, resource-intensive, and associated with patient discomfort and procedural risks. Noninvasive alternatives, such as fecal occult blood tests and fecal immunochemical tests, are more accessible but have suboptimal sensitivity, particularly for early-stage tumors, and are prone to false positives from noncancer-related bleeding.^11–19^ These shortcomings underscore the need for complementary noninvasive biomarkers with improved diagnostic performance.

Blood-based molecular biomarkers offer advantages in accessibility, repeatability, and objectivity.^20,21^ Conventional CRC markers such as carcinoembryonic antigen (CEA) and carbohydrate antigen 19-9 (CA19-9) are helpful for monitoring disease progression and treatment response^22–24^ but lack the sensitivity and specificity for primary screening or early detection. This underscores the ongoing need for new biomarker discovery strategies that identify more reliable indicators of CRC.

Metabolomics has emerged as a valuable tool for biomarker discovery in cancer and other diseases. As the downstream readout of genomic, transcriptomic, and proteomic processes, the metabolome reflects functional, physiological, and pathological states.^25–28^ Advances in liquid chromatography–mass spectrometry (LC–MS) have enabled comprehensive profiling of small-molecule metabolites in biofluids with high sensitivity and specificity. Both untargeted and targeted metabolomics approaches have been successfully applied to identify potential biomarkers and elucidate mechanisms in CRC.^29–31^

Integrating machine learning with metabolomics data analysis enables the handling of high-dimensional data sets and improves feature selection and pattern recognition.^32^ Random forest algorithms, in particular, are widely used in metabolomics studies because of their robustness to overfitting and their ability to rank feature importance.^33–35^ Random forest algorithms have demonstrated significant utility in analyzing metabolomic data by constructing multiple decision trees and aggregating their predictions, thereby reducing overfitting and enhancing generalization across diverse patient populations.

While fecal immunochemical tests (FIT) and serum carcinoembryonic antigen (CEA) remain widely used in colorectal cancer (CRC) screening and surveillance, both approaches have recognized limitations. FIT performance is affected by intermittent bleeding and reduced sensitivity for early-stage diseases, and CEA lacks sufficient sensitivity and specificity for population-level screening. Emerging blood-based assays, including circulating tumor DNA and methylation-based panels, offer promising advances but often require complex workflows and remain costly or inaccessible in routine clinical settings. In this context, plasma metabolomics is a complementary strategy that captures systemic metabolic alterations associated with the tumor presence and host–microbiome interactions. Given that colorectal carcinogenesis is often accompanied by significant gut microbiome dysbiosis and cachexia-associated metabolic reprogramming, we hypothesized that systematic depletion of host-microbial metabolites could serve as a sensitive marker for early disease detection. Rather than replacing existing screening tools, metabolite-based biomarkers may provide additional biological information to enhance risk stratification and support clinical decision-making.

In this study, we applied an integrated multiplatform metabolomics workflow that combines untargeted and targeted LC–MS analyses with machine learning to analyze plasma samples from CRC patients and healthy controls. We aimed to identify and validate a panel of plasma metabolite biomarkers with potential utility for noninvasive CRC detection. We also assessed the phenotypic effects of selected metabolites to gain insights into their biological relevance in CRC.

## 2 Results

### 2.1 Feature Selection of Metabolites using the Random Forest Algorithm and Receiver Operating Characteristic Analysis

To identify differential metabolites between CRC patients and healthy individuals, we analyzed plasma samples from two independent cohorts. The discovery cohort from NCKUH comprised 172 CRC patients and 115 healthy volunteers. In comparison, an independent validation cohort from E-Da Hospital included 47 CRC patients and 47 healthy volunteers (detailed clinical characteristics are presented in Table 1). Our comprehensive workflow for metabolite biomarker identification using LC–MS coupled with machine learning is illustrated in Figure 1. Raw high-resolution mass spectrometry data underwent systematic processing, including retention time alignment and signal intensity normalization relative to internal standards. This rigorous processing yielded 146,880 distinct spectral features across all samples. To eliminate subjective bias in feature selection, the random forest model used 20 trees, selected after comparing performance with 50 and 100 trees, which yielded similar accuracy (differences <1%) but higher computational costs (Figure S1).

**Table 1:** Demographic and Clinical Characteristics of Study Participants in Discovery and Validation Cohorts^a^

		discovery group		validation group	
		healthy volunteer	CRC patient	healthy volunteer	CRC patient
	total	115	172	47	47
gender	male	56	93	17	24
	female	59	79	30	23
age	<60	107	41	42	21
	≥60	8	127	5	26
	mean ± standard deviation (range)	40 ± 12.08 (24–68)	71 ± 9.47 (29–93)	34 ± 12.54 (22–66)	61 ± 11.39 (30–88)
	median	37	72	30	61
stage	I	-	28	-	11
	II	-	47	-	9
	III	-	60	-	17
	IV	-	37	-	10
primary site	colon	-	141	-	12
	rectal	-	27	-	18
	other	-	4	-	17

a Comparison of demographic features and clinical parameters between colorectal cancer patients and healthy controls across both study cohorts. Data are presented as mean ± standard deviation or number of cases (percentage) as appropriate.

**Figure 1:**
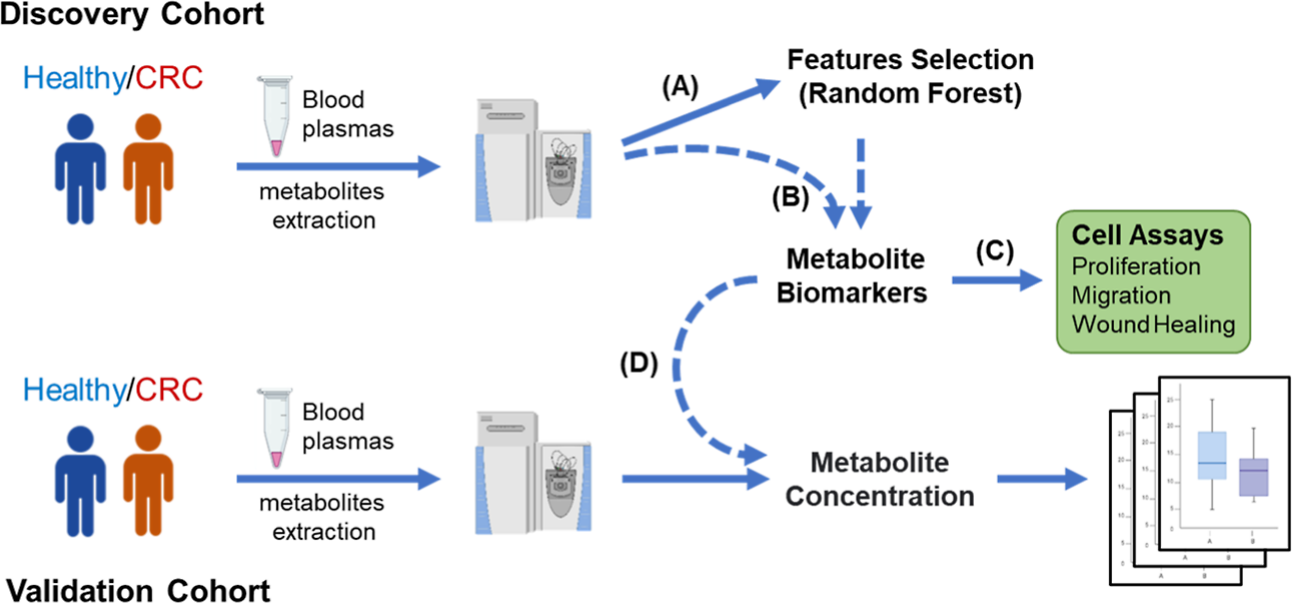
Multiplatform metabolomics workflow for discovery and validation of colorectal cancer biomarkers. The comprehensive pipeline outlines a systematic approach to biomarker development: (A) discovery stage integrating high-resolution mass spectrometry with a random forest algorithm for unbiased feature selection from CRC and control sera; (B) target analysis phase using ROC curve assessment and database annotation to identify clinically relevant metabolite candidates; (C) functional validation establishing biological relevance through cell-based assays assessing metabolites’ effects on CRC cell migration, invasion, and proliferation; (D) clinical validation confirming diagnostic utility through quantification and statistical analysis in an independent patient cohort. This integrated workflow combines machine learning-based feature selection, quantitative metabolomics, and phenotypic effects to identify and validate robust metabolite biomarkers with potential for early CRC detection and biological significance.

### 2.2 Robust Prioritization of CRC-Associated Metabolite Features Using Random Forest Analysis

Given the high dimensionality of untargeted LC–MS metabolomics data, our objective was not to build a single optimized black-box classifier but rather to prioritize metabolite features that consistently distinguish CRC patients from healthy controls across multiple sampling partitions.

Using Random Forest analysis applied independently to ten randomly partitioned feature subsets, we identified 1007 features that reproducibly discriminated between CRC and control samples. Internal validation within the discovery cohort showed strong classification performance (accuracy of 97.7%), confirming that the selected feature set retained substantial discriminatory information (Figure 2A). This performance reflects feature-set separability rather than final diagnostic modeling. Orthogonal partial least-squares-discriminant analysis further demonstrated clear separation between CRC and healthy plasma metabolomic profiles based on these features (Figure 2B), and permutation testing confirmed model robustness (Figure 2C).

**Figure 2:**
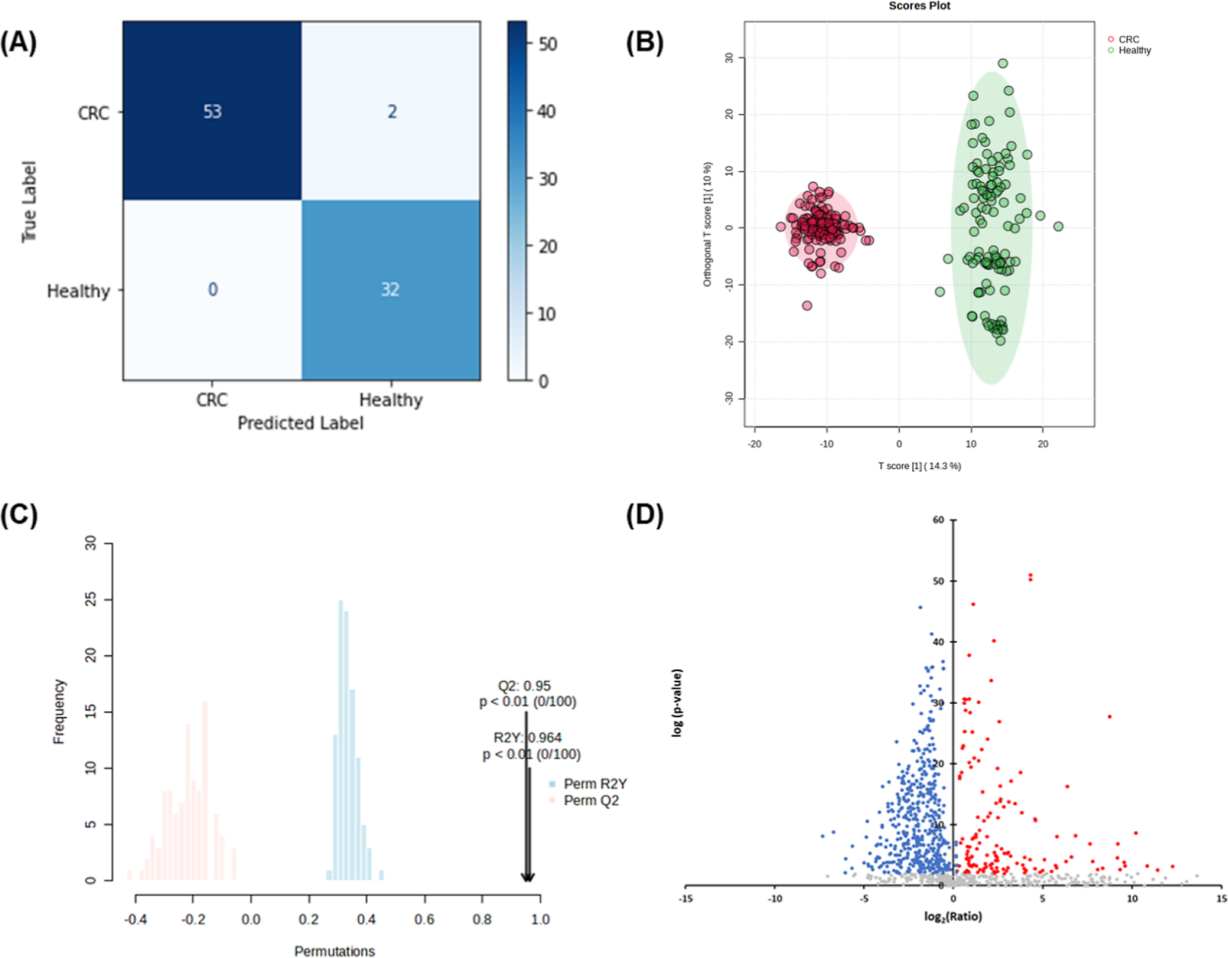
Machine learning-based feature selection for CRC biomarker discovery. (A) Confusion matrix evaluating the performance of features selected by the random forest algorithm. Model validation on 30% of the discovery cohort samples (*N* = 87) achieved a 97.7% accuracy and a 98.1% F1 score, demonstrating robust classification performance. (B) OPLS-DA score plot showing distinct metabolic profiles of the 1007 features in CRC patients (red) and healthy individuals (green) in the discovery cohort. (C) Permutation testing results across 100 permutations with *p* < 0.01, yielding cumulative R^2^Y and Q^2^ values of 0.964 and 0.95, respectively, confirming model validity. (D) Volcano plot illustrating the significance and fold-change distribution of the 1007 selected features via random forest analysis, highlighting 277 upregulated (red dots) and 543 downregulated (blue dots) features in CRC patients compared with healthy controls.

Subsequent volcano plot analysis showed that the majority of discriminatory features were downregulated in CRC plasma samples, suggesting a global metabolic suppression associated with disease status (Figure 2D). To prioritize features with the most significant translational potential, ROC analysis was performed, and features with an AUC >0.90 were retained for metabolite annotation. This process yielded 18 high-confidence metabolite annotations, from which five commercially available metabolites were selected for quantitative validation and phenotypic effects. We also employed 5-fold cross-validation during random forest model training on the discovery cohort to optimize hyperparameters, assess internal validity, and prevent overfitting.

### 2.3 Validation of Machine-Learning-Selected Metabolite Candidates for CRC Detection

For validation and further characterization, we selected five commercially available annotated metabolites: *N*-methylcytisine, 2-piperidone, theophylline, dl-norleucine, and linolenic acid. Initial ROC curve analysis in the discovery cohort revealed exceptional diagnostic potential for four of these metabolites, with AUC values exceeding 0.95 for all except dl-norleucine (Figure S2). To ensure accurate quantification, we established concentration standard curves to measure these compounds in both the discovery and validation cohorts. The diagnostic performance of these five metabolites was rigorously evaluated in the independent validation cohort using scatter plots and ROC curve analyses (Figure 3A–E). Notably, the concentrations of all five metabolites were significantly lower in CRC patients than in healthy controls. This consistent pattern across both the discovery and validation cohorts strengthens their potential as reliable CRC biomarkers.

**Figure 3:**
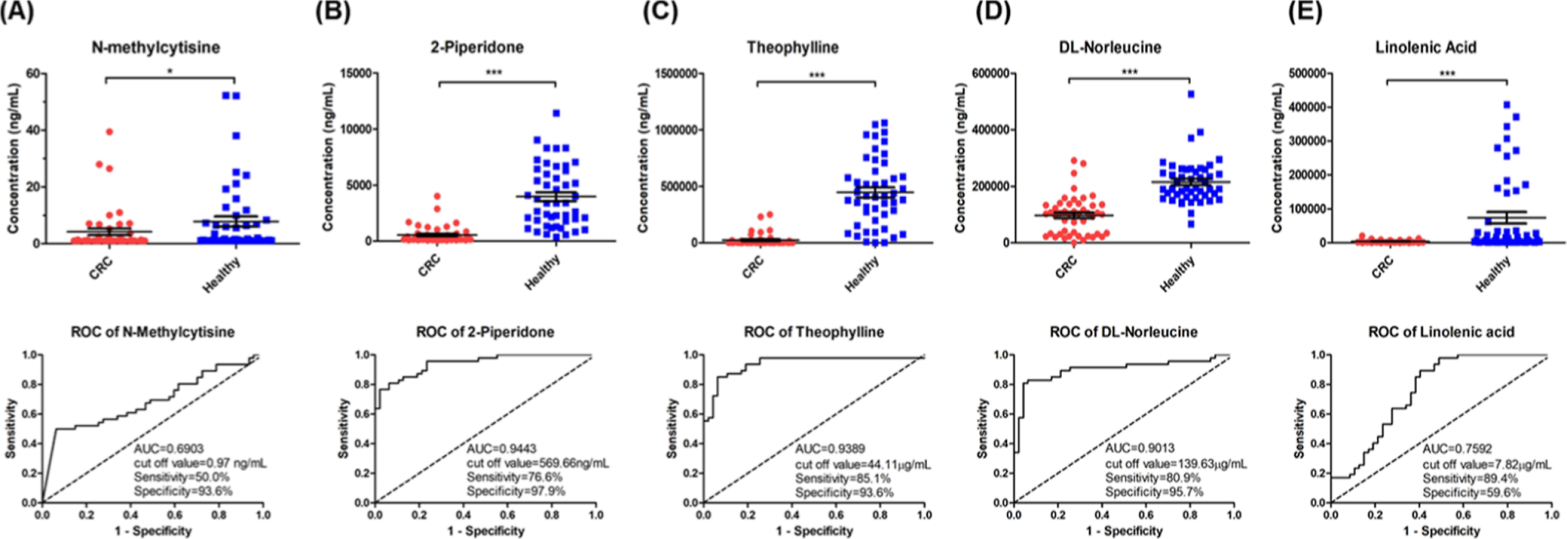
Comparison of candidate metabolite levels between CRC patients and healthy controls in the validation cohort. Upper panel: Scatter dot plots showing differential plasma levels of (A) *N*-methylcytisine, (B) 2-piperidone, (C) theophylline, (D) dl-norleucine, and (E) linolenic acid between CRC patients (*n* = 47) and healthy controls (*n* = 47) in the validation cohort. *P*-values indicate statistical significance between groups. Lower panel: Corresponding ROC curves assessing diagnostic performance of each metabolite, with AUC values of 0.6903, 0.9443, 0.9389, 0.9013, and 0.7592, respectively, demonstrating high sensitivity and specificity for CRC detection. All five metabolites showed significantly lower levels in CRC patients than in healthy controls.

### 2.4 Robustness of Candidate Metabolites across Demographic and Clinical Subgroups

We further analyzed the concentrations of these metabolites across different CRC stages, as detailed in Table 2. Except for *N*-methylcytisine, which showed a relatively lower AUC value in the validation cohort, the other four metabolites exhibited consistently reduced concentrations across all CRC stages compared with healthy controls. Notably, correlation analysis between metabolite levels and cancer progression revealed that dl-norleucine concentration decreased progressively with advancing cancer stage (Figure 4A–E), suggesting its potential as a staging biomarker.

**Table 2:** Quantification of Candidate Metabolites across Different CRC Stages in the Validation Cohort^a^

metabolite	concentrations		AUC	*p*-value	cut-off value	sensitivity	specificity	accuracy
	CRC patient	healthy volunteer						
*N*-methylcytisine	4.15 ± 1.15 ng/mL	7.82 ± 1.81 ng/mL	0.6903	0.0464	0.97 ng/mL	50.0%	93.6%	71.8%
2-piperidone	542.14 ± 111.00 ng/mL	3963.58 ± 397.87 ng/mL	0.9443	<0.0001	569.66 ng/mL	76.6%	97.9%	87.3%
theophylline	23.00 ± 8.04 μg/mL	446.40 ± 44.08 μg/mL	0.9389	<0.0001	44.11 μg/mL	85.1%	93.6%	89.4%
dl-norleucine	97.05 ± 10.03 μg/mL	215.38 ± 113.00 μg/mL	0.9013	<0.0001	139.63 μg/mL	80.9%	95.7%	88.3%
linolenic acid	3.91 ± 0.55 μg/mL	73.88 ± 17.00 μg/mL	0.7592	<0.0001	7.82 μg/mL	89.4%	59.6%	74.5%

a Plasma concentrations of the five identified metabolites across different CRC stages (I–IV) compared to healthy controls. Data represent the mean ± standard deviation, with statistical significance indicated by *p*-values from appropriate statistical tests.

**Figure 4:**
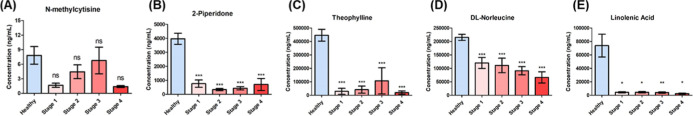
Stage-dependent alterations in candidate metabolite concentrations during CRC progression. Plasma concentrations of (A) *N*-methylcytisine, (B) 2-piperidone, (C) theophylline, (D) dl-norleucine, and (E) linolenic acid, stratified by CRC stage (I through IV), are compared with those in healthy controls. Scatter plots with horizontal lines representing mean values show stage-specific concentration patterns for each metabolite. dl-Norleucine exhibits a statistically significant progressive decrease with advancing cancer stages (*p* < 0.05), highlighting its potential as a staging biomarker. All metabolites show significantly lower concentrations in CRC patients than in healthy controls, with stage-dependent alterations that may reflect underlying disease mechanisms.

To assess whether demographic factors confounded the identified metabolite alterations, we examined plasma metabolite concentrations by age, sex, tumor location, and disease stage. Importantly, four of the five metabolites (2-piperidone, theophylline, dl-norleucine, and linolenic acid) remained significantly reduced in CRC patients compared with healthy controls in both early-stage (I–II) and late-stage (III–IV) diseases (Figure 5A), indicating that their discriminatory capacity is not limited to advanced cancer.

**Figure 5:**
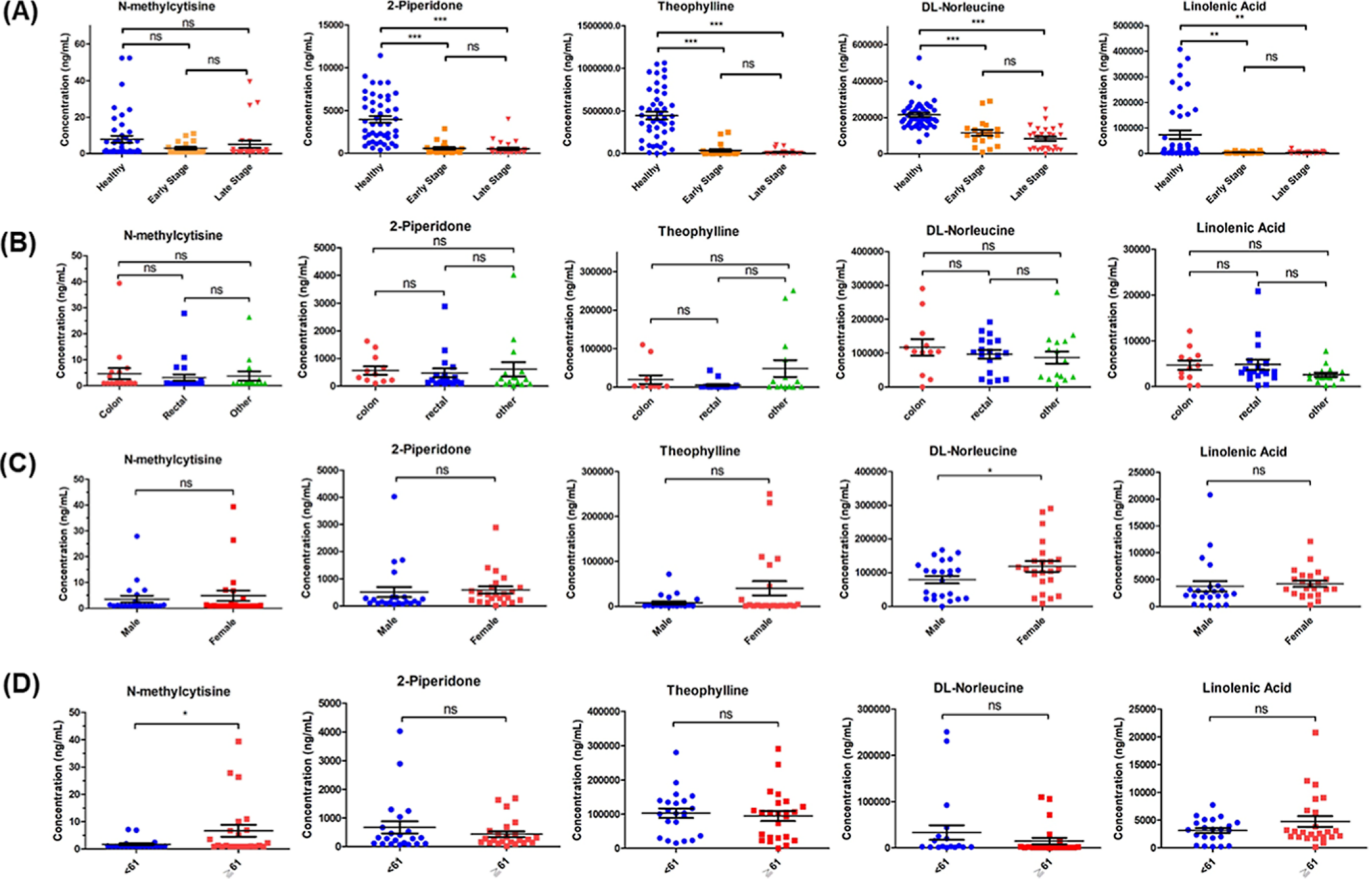
Comparative analysis of candidate metabolite levels across clinical subgroups in CRC patients. Scatter plots show the distribution of the five identified metabolites stratified by (A) disease progression—comparing healthy controls with early-stage (I–II) and late-stage (III–IV) CRC patients. Four metabolites (except *N*-methylcytisine) showed significantly lower levels in both early- and late-stage CRC compared with healthy controls, with no significant difference between the two stages. (B) Primary tumor site—analysis across anatomical locations revealed no significant differences in metabolite concentrations. (C) Gender distribution—dl-norleucine was the only metabolite showing significant differences in concentration between male and female CRC patients (*p* = 0.0248). (D) Age stratification—using the median age (61 years) as a cutoff, only *N*-methylcytisine demonstrated significant concentration differences between age groups (*p* = 0.0160). Horizontal lines represent mean values, and asterisks indicate statistical significance (*p < 0.05, **p < 0.01, ***p < 0.001).

Age-stratified analysis using the median cohort age as a cutoff showed that most metabolites exhibited consistent concentration patterns across age groups with no significant age-dependent differences observed for 2-piperidone, theophylline, dl-norleucine, or linolenic acid in CRC patients (Figure 5B). In contrast, *N*-methylcytisine showed modest age-associated variation, suggesting that age-related metabolic factors may partially influence its diagnostic contribution. Sex-based stratification revealed minimal impact on metabolite concentrations, except for dl-norleucine, which showed a modest but statistically significant difference between male and female CRC patients (Figure 5C). No significant differences were observed when stratifying by the primary tumor location (Figure 5D). Collectively, these analyses indicate that the majority of metabolites in the five-metabolite panel demonstrate demographic robustness, supporting their potential utility as CRC-associated biomarkers across heterogeneous patient populations.

Given the significant age difference between the CRC patients (71 ± 9 years) and healthy controls (40 ± 12 years) in the discovery cohort, we sought to verify that the observed reductions in metabolite levels were not driven by aging-related physiology. In our multivariate logistic regression model adjusted for age, four of the five metabolites (2-piperidone, theophylline, dl-norleucine, and linolenic acid) remained statistically significant (*p* < 0.05), confirming their status as independent predictors of CRC (Figure S3). Consistent with its lack of age dependence in healthy controls, theophylline remained significantly associated with CRC status after adjustment for age and sex (Figure S4). *N*-Methylcytisine showed a partial attenuation in predictive power after age adjustment, consistent with our stratified analysis (Figure 5B), which indicated a potential age-associated variation. Furthermore, when we analyzed the healthy control group in isolation, no significant correlation was found between age and the plasma levels of 2-piperidone, theophylline, dl-norleucine, or linolenic acid, suggesting these are stable markers unrelated to normal chronological aging in the absence of disease.

### 2.5 Integrated Five-Metabolite Panel Improves Diagnostic Accuracy for CRC Detection

While individual metabolites demonstrated diagnostic potential, we hypothesized that a multimarker approach would provide a more robust and comprehensive assessment of CRC status. Quantification in the validation cohort confirmed that all five machine-learning-selected metabolites (*N*-methylcytisine, 2-piperidone, theophylline, dl-norleucine, and linolenic acid) exhibited significantly lower concentrations in CRC patients than in healthy controls. Notably, three of these metabolites demonstrated exceptional discriminatory capability with AUC values exceeding 0.9 (Figure 4 and Table 2). To develop an integrated diagnostic model, we employed logistic regression analysis to combine information from all five metabolites. This approach allowed us to determine the relationship between metabolite concentrations and the probability of CRC presence by leveraging the complementary information from each biomarker. The model coefficients were derived from the training set and then applied to predict CRC probability in individual cases based on their specific metabolite profiles.

The performance of this logistic regression model was assessed using a confusion matrix to compare predicted classifications against actual clinical diagnoses (Figure 6A). When evaluated on the test set (comprising 30% of the validation cohort, *N* = 29), the model achieved an accuracy of 86.2%, demonstrating its effectiveness in estimating the CRC probability. More comprehensive evaluation of the entire validation cohort revealed that the five-metabolite panel achieved an impressive AUC of 0.9679 (95% CI, 0.9250–0.999), with a 97.9% sensitivity (95% CI, 88.9%–99.9%), an 89.4% specificity (95% CI, 76.9%–96.5%), and a 93.7% overall accuracy (95% CI, 86.2%–98.1%) (Figure 6B). Importantly, this integrated metabolite panel substantially outperformed any individual metabolite for CRC detection, confirming the value of a multimarker approach. The high sensitivity (97.9%) is particularly noteworthy, as it indicates the panel’s ability to correctly identify CRC cases with minimal false negatives, which is crucial for an effective screening tool. Together, these results demonstrate that our machine-learning-derived metabolite panel offers a promising approach to noninvasive CRC detection, with potential applications in screening and diagnosis.

**Figure 6:**
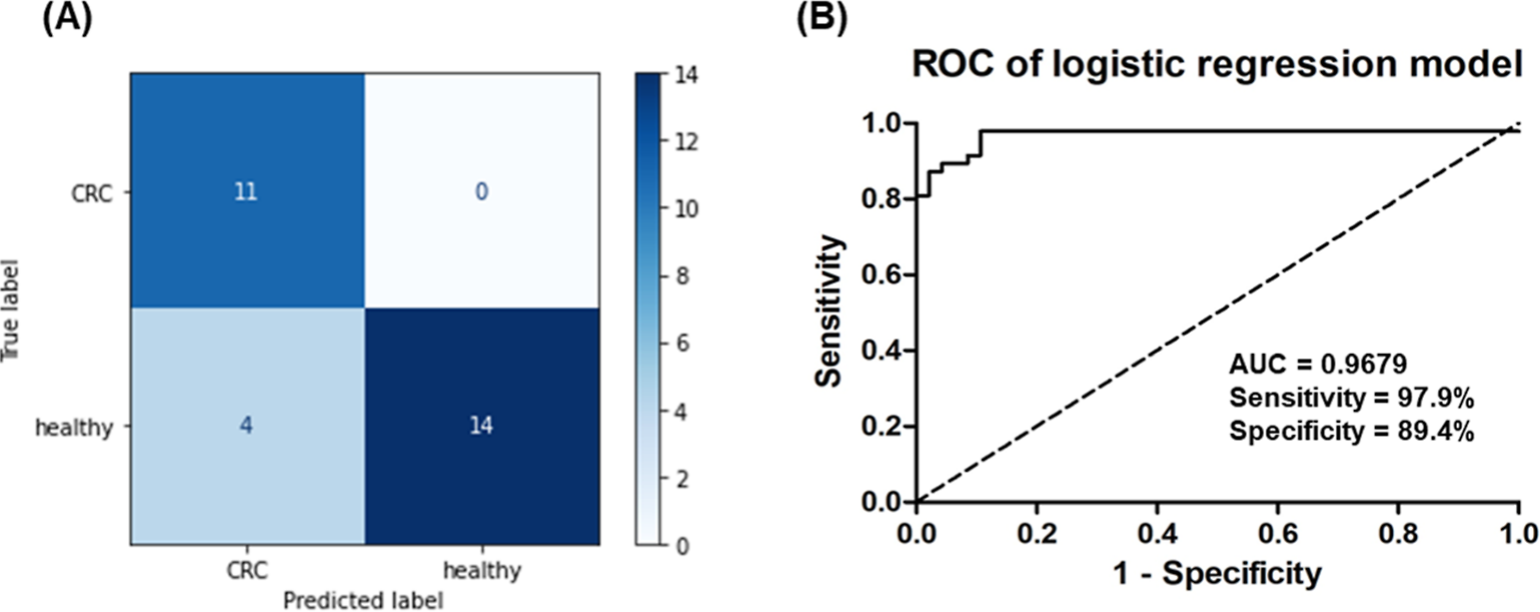
Multimarker panel performance for CRC detection. (A) Confusion matrix for the logistic regression model incorporating all five metabolites, achieving an 86.2% accuracy on the test set (30% of the validation group, *N* = 29). (B) ROC curve analysis of the five-metabolite panel shows an AUC of 0.9679 (95% CI: 0.9250–0.999) with an 89.4% specificity and a 97.9% sensitivity, demonstrating superior performance compared with individual metabolites for CRC detection.

### 2.6 Phenotypic Effects of CRC-Associated Metabolites on CRC Cell Proliferation and Motility

To explore the biological relevance of the CRC-associated metabolites identified in our plasma metabolomics analysis, we evaluated their effects on cancer cell phenotypes associated with tumor progression, including proliferation, migration, and wound closure, using the HCT116 and T5088 colorectal cancer cell lines.

At concentrations comparable to those observed in healthy donor plasma, none of the five metabolites significantly altered HCT116 cell proliferation, indicating minimal cytotoxicity under physiologically relevant conditions (Figure 7A). In contrast, T5088 cells showed dose-dependent sensitivity to higher concentrations of *N*-methylcytisine and theophylline, suggesting cell-line-specific metabolic responsiveness rather than a generalized antiproliferative effect (Figure 7B).

**Figure 7:**
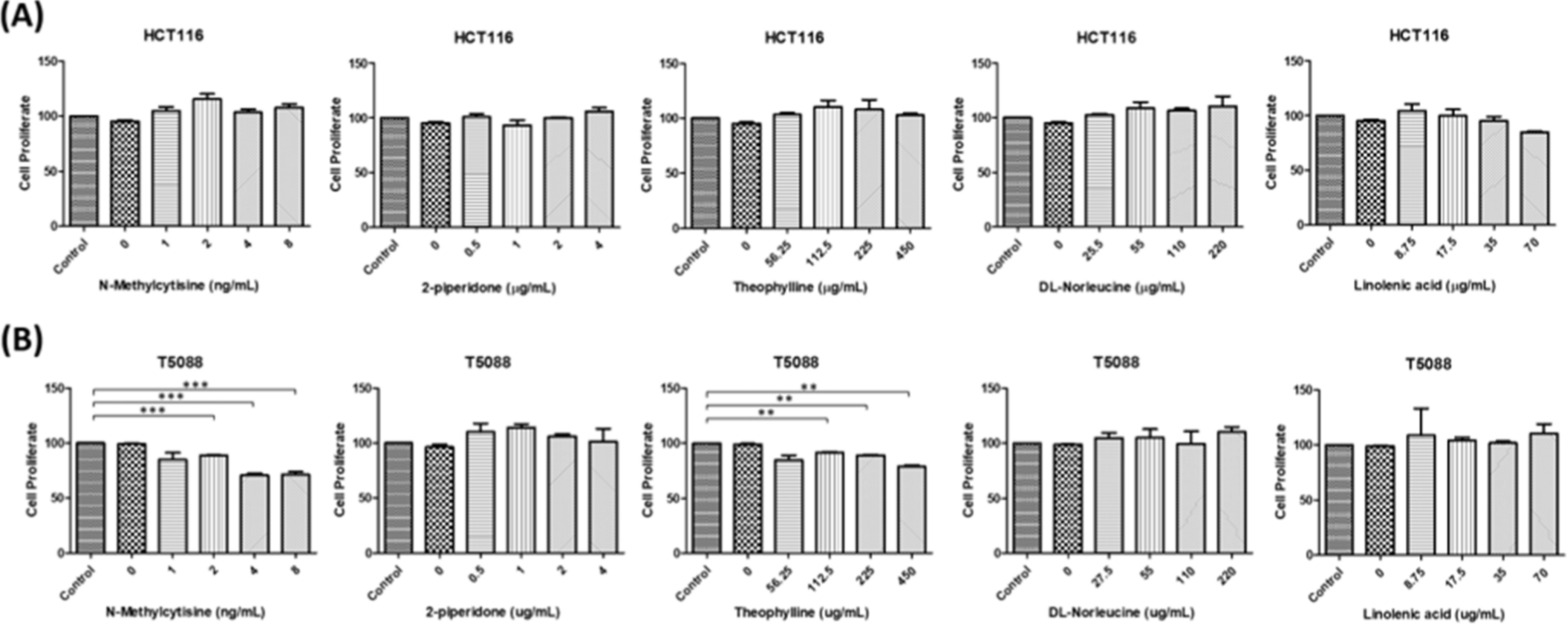
Effects of candidate metabolites on colorectal cancer cell proliferation. Dose-dependent CCK-8 proliferation assays evaluated the potential cytotoxic effects of the five identified metabolites on (A) HCT116 and (B) T5088 CRC cell lines. Cells were treated with increasing concentrations of *N*-methylcytisine, 2-piperidone, theophylline, dl-norleucine, and linolenic acid for 24 h, spanning physiologically relevant levels to higher experimental doses. Results are shown as cell viability as a percentage of control. While physiologically relevant concentrations had minimal effects on HCT116 proliferation, *N*-methylcytisine and theophylline exhibited notable dose-dependent cytotoxicity against T5088 cells, suggesting cell line-specific responses to these metabolites.

While effects on proliferation were limited, our investigations revealed more pronounced impacts on cancer cell invasion and metastatic capacity. Using Transwell migration assays, we found that treatment with 2-piperidone (4 μg/mL), theophylline (450 μg/mL), dl-norleucine (220 μg/mL), and linolenic acid (70 μg/mL) significantly suppressed the migration ability of HCT116 cells compared to control conditions (Figure 8A). Similarly, *N*-methylcytisine (8 ng/mL), 2-piperidone (4 μg/mL), and theophylline (450 μg/mL) markedly inhibited the migration of T5088 cells (Figure 8B), further supporting the modulation of migration-related phenotypes of these metabolites. Complementary wound-healing assays corroborated these findings. All five metabolites demonstrated inhibitory effects on HCT116 cell migration, with quantifiably reduced wound closure compared with controls (Figure 9A). The effects were more selective in T5088 cells, where theophylline exhibited the strongest migration inhibition, while *N*-methylcytisine and linolenic acid showed moderate but statistically nonsignificant inhibitory effects. 2-Piperidone and dl-norleucine had a negligible impact on T5088 wound healing (Figure 9B).

**Figure 8:**
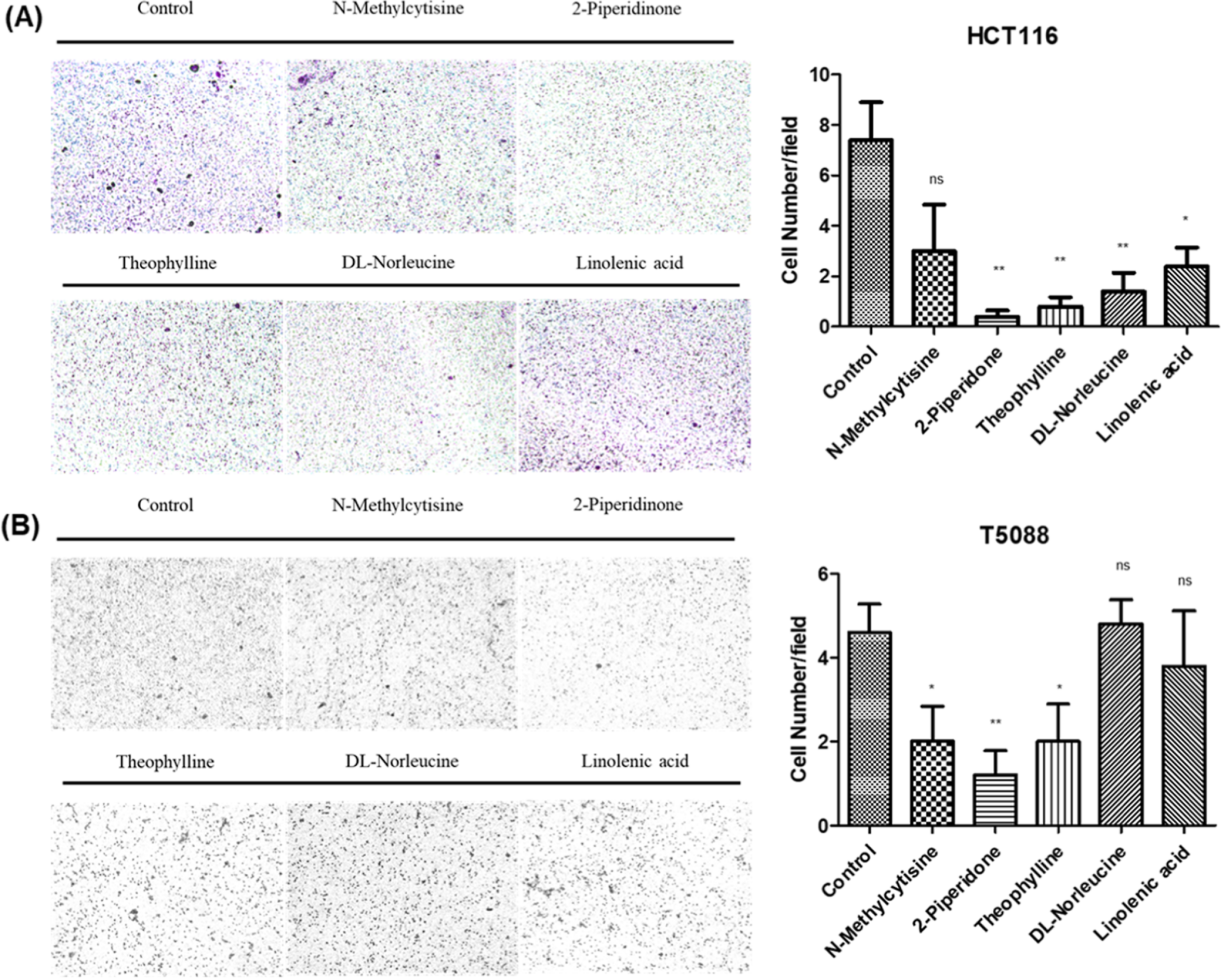
Impact of candidate metabolites on CRC cell migration. Transwell migration assay evaluating the functional effects of identified metabolite biomarkers on cancer cell motility. (A) HCT116 and (B) T5088 CRC cell lines were treated with physiologically relevant concentrations of *N*-methylcytisine (8 ng/mL), 2-piperidone (4 μg/mL), theophylline (450 μg/mL), dl-norleucine (220 μg/mL), and linolenic acid (70 μg/mL) for 18 h. Left panels: Representative photomicrographs of crystal violet-stained migrated cells. Right panels: Quantitative analysis of migrated cells expressed as a percentage of control. In HCT116 cells, 2-piperidone, theophylline, dl-norleucine, and linolenic acid significantly inhibited migration, whereas *N*-methylcytisine, 2-piperidone, and theophylline demonstrated significant antimigratory effects in T5088 cells. Statistical significance is indicated as *p < 0.05, **p < 0.01, and ***p < 0.001 compared to control. These findings suggest that decreased levels of these metabolites in CRC patients may contribute to enhanced modulation of migration-related phenotypes.

**Figure 9:**
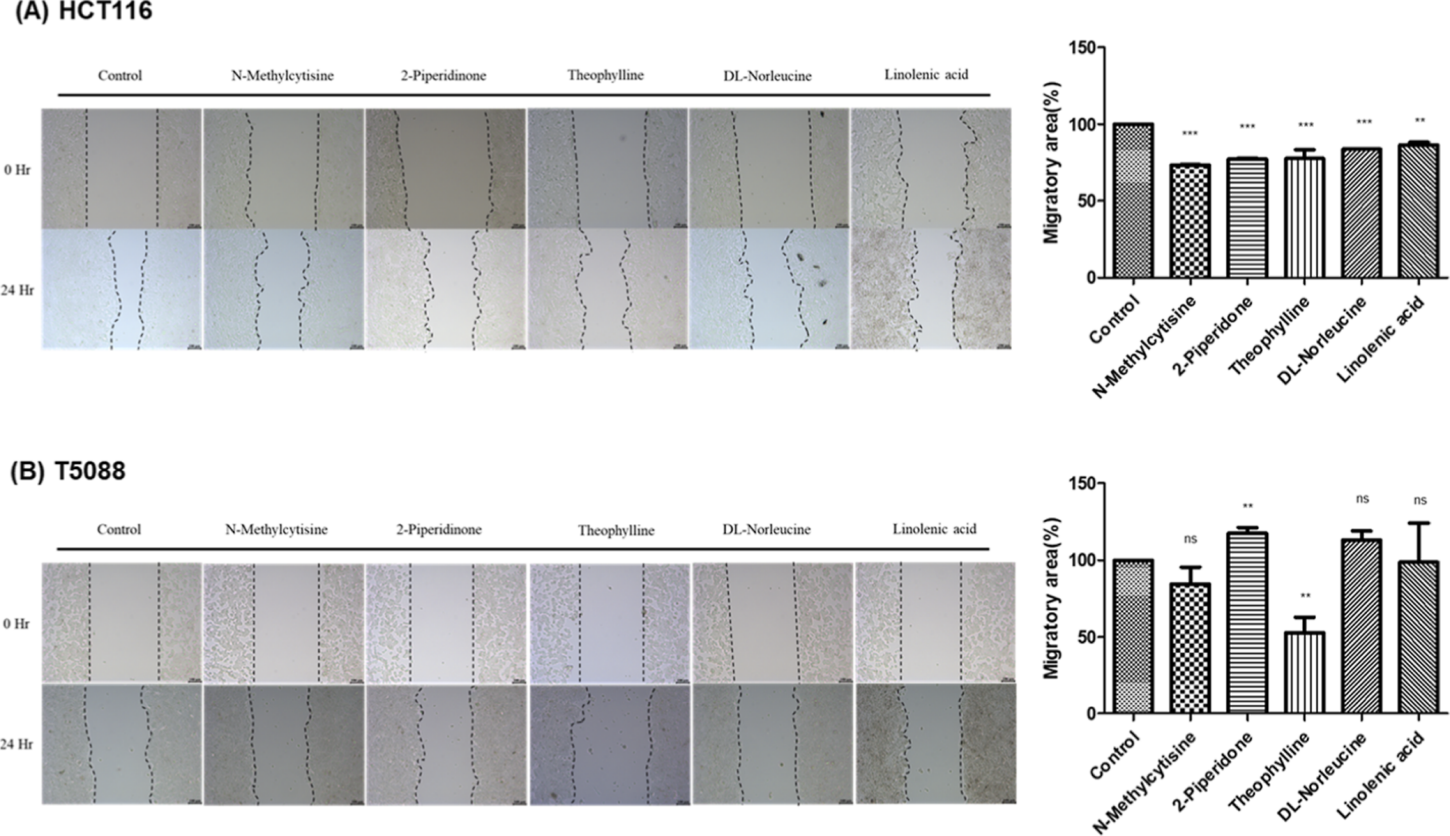
Effects of candidate metabolites on CRC cell motility assessed by the wound-healing assay. Scratch-wound healing assay evaluating the impact of identified plasma metabolite biomarkers on cancer cell migration. (A) HCT116 and (B) T5088 CRC cell lines were treated with physiologically relevant concentrations of *N*-methylcytisine (8 ng/mL), 2-piperidone (4 μg/mL), theophylline (450 μg/mL), dl-norleucine (220 μg/mL), and linolenic acid (70 μg/mL) for 24 h. Representative photomicrographs show wound closure at 0 and 24 h postscratch. Quantitative analyses of wound closure rates for HCT116 and T5088 cells are expressed as a percentage of control. In HCT116 cells, all five metabolites significantly inhibited wound closure and migration, whereas in T5088 cells, only theophylline demonstrated significant inhibition, with *N*-methylcytisine and linolenic acid showing moderate but statistically nonsignificant effects. 2-Piperidone and dl-norleucine had minimal impact on T5088 migration. Statistical significance is indicated as *p < 0.05, **p < 0.01, and ***p < 0.001 compared to untreated control. These results, combined with the Transwell assay findings, reinforce the potential biological significance of these metabolites in regulating CRC metastatic behavior, suggesting that reduced levels in CRC patients may contribute to enhanced tumor invasiveness.

Collectively, these findings indicate that multiple metabolites reduced in CRC patient plasma retain biological activity capable of modulating cancer cell motility-related phenotypes in vitro under physiologically relevant conditions, providing a biological context for the metabolomic alterations observed without establishing direct mechanistic or causal relationships between circulating metabolite levels and tumor behavior in vivo.

## 3 Discussion

Several methodological considerations are relevant in interpreting the machine learning component of this study. Rather than pursuing maximal predictive performance with complex or opaque algorithms, we adopted a conservative strategy that emphasizes feature robustness, biological interpretability, and translational relevance. Random forest analysis was used solely for feature prioritization in the high-dimensional metabolomics data. The final diagnostic model relied on logistic regression with quantified metabolites measured in an independent cohort. This design minimizes the risk of overfitting by separating feature discovery from classifier construction and avoiding repeated model optimization on the validation data. While alternative machine learning approaches could potentially yield marginal gains in classification performance, such gains often come at the cost of reduced interpretability and increased risk of data set-specific bias. In contrast, our approach prioritizes metabolite signatures that are reproducible, biologically plausible, and compatible with future clinical assay development.

Our metabolomics analysis identified five metabolites, *N*-methylcytisine, 2-piperidone, theophylline, norleucine, and linolenic acid, that consistently exhibited lower concentrations in CRC patients compared with healthy controls. These findings, coupled with our functional studies demonstrating their inhibitory effects on cancer cell invasion and migration, provide new insights into CRC pathogenesis and potential biomarkers for early detection.

*N*-Methylcytisine, a natural compound found in plants such as *Thermopsis lanceolata* and *Sophora chrysophylla*, has demonstrated diverse pharmacological properties, including analgesic, antidiabetic, and anti-inflammatory effects.^36,37^ In traditional Chinese medicine, *N*-methylcytisine is a component of the herbal compound Kushen, which has been used for treating nonsmall cell lung cancer.^38,39^ Our study provides the first evidence of its potential role in CRC detection, as demonstrated by reduced levels in CRC patients compared to healthy controls. Furthermore, our functional assays revealed inhibitory effects on CRC cell migration, suggesting potential biological relevance beyond its biomarker utility.

2-Piperidone (δ-valerolactam), a derivative of piperidine, has been previously reported at lower levels in the plasma of ovarian cancer patients,^40–43^ consistent with our findings in CRC patients. Importantly, 2-piperidone is produced by gastrointestinal bacteria,^44,45^ establishing a connection to the gut microbiome, which plays an established role in colorectal carcinogenesis. The inhibitory effect of 2-piperidone on CRC cell migration and invasion observed in our functional studies suggests a potential protective mechanism that may be compromised in CRC patients with reduced levels of this metabolite.

Theophylline (1,3-dimethylxanthine), commonly used as a bronchodilator for respiratory conditions,^46^ has demonstrated antiproliferative effects in CRC and breast cancer cells in previous studies.^47–49^ Recent studies have developed algorithms to uncover novel applications of theophylline in cancer therapy.^46–48^ The identification of theophylline as a downregulated marker requires careful biological interpretation. While theophylline is clinically used as a bronchodilator, circulating theophylline in the general population primarily results from hepatic metabolism of dietary caffeine by the cytochrome P450 enzyme CYP1A2. Consequently, the observed reduction in CRC patients could reflect reduced dietary intake of methylxanthines (coffee and tea) due to disease-associated changes in appetite. However, we observed decreased theophylline levels consistently even in early stage (Stage I) patients, who typically do not exhibit the profound anorexia or ‘sickness behavior’ seen in advanced malignancy. Alternatively, this reduction may reflect systemic metabolic reprogramming. CYP1A2 activity is downregulated by systemic inflammation and cytokines (such as IL-6), which are elevated in CRC. Therefore, the lower plasma theophylline levels likely serve as a surrogate marker for host metabolic immune suppression or alterations in the gut microbiome’s capacity to metabolize methylxanthines, rather than solely reflecting dietary habits.

Norleucine (2-aminohexanoic acid), an isomer of leucine previously detected in human blood,^42,43^ showed the most interesting stage-dependent pattern in our analysis. The progressive decline in norleucine concentrations with advancing CRC stages suggests its potential as a staging biomarker, which could complement existing methods for disease monitoring. The association of norleucine with intestinal bacteria^45^ further strengthens the connection between the gut microbiome and CRC pathogenesis. Our novel observation that norleucine inhibits cancer cell migration provides a functional context for its potential role in CRC progression.

Linolenic acid, an essential omega-3 fatty acid synthesized by bacteria, has been reported to inhibit cancer cell proliferation, adhesion, and invasion in various cancer types.^50^ Recent studies have provided direct evidence for its antimetastatic properties in osteosarcoma.^51^ Our findings in CRC cells corroborate these antimetastatic effects and suggest that reduced linolenic acid levels in CRC patients may contribute to increased tumor invasiveness. This represents another link between altered bacterial metabolism and CRC progression.

The phenotypic assays in this study were designed to assess whether CRC-associated plasma metabolites exhibit biological activity relevant to cancer cell behavior rather than to establish direct molecular mechanisms. Several metabolites reduced in CRC plasma modulated migration-related phenotypes in vitro, supporting the notion that these metabolites may reflect systemic metabolic states that influence tumor permissiveness. It is important to emphasize that plasma metabolites may serve as indicators or mediators of host–tumor–microbiome interactions rather than as direct tumor-suppressive agents. The observed phenotypic effects should not be interpreted as evidence that restoring circulating metabolite levels would directly inhibit tumor progression. Instead, these findings provide biological plausibility that the identified metabolites are not merely statistical markers but are components of metabolic environments associated with altered tumor cell behavior. Moreover, the heterogeneity observed among CRC cell lines underscores the context-dependent nature of metabolite responsiveness, further supporting a model in which circulating metabolite levels reflect broader systemic or microbiome-driven influences rather than uniform tumor-intrinsic mechanisms. These in vitro findings require in vivo confirmation to establish mechanisms.

The identification of three bacteria-associated metabolites (2-piperidone, norleucine, and linolenic acid) in our panel highlights the importance of the microbiome–metabolome axis in CRC. Given the growing evidence of gut dysbiosis in CRC pathogenesis, these metabolites may serve as functional intermediaries linking altered microbial communities to cancer development. These connections are speculative without paired microbiome data and warrant further investigation, particularly regarding whether these metabolic alterations are consequences or contributors to CRC pathogenesis.

From a clinical perspective, our five-metabolite panel demonstrated robust diagnostic performance (a 93.7% accuracy, a 97.9% sensitivity, and an 89.4% specificity) in an independent validation cohort. The high sensitivity is particularly relevant for screening applications where minimizing false negatives is critical. Notably, the panel maintained discriminatory power for early-stage CRC detection, addressing a significant clinical need for noninvasive screening alternatives to colonoscopy.

Several methodological aspects of our study merit consideration. The integration of untargeted metabolomics with machine learning-based feature selection provided an unbiased approach to biomarker discovery, reducing the risk of overlooking significant metabolic alterations. The validation in an independent cohort strengthens the reliability of our findings, although larger multicenter studies are needed to establish a broader clinical applicability.

The diagnostic performance of the five-metabolite panel should be interpreted in the context of existing CRC screening and blood-based biomarkers. Serum CEA, while useful for disease monitoring, has limited sensitivity for early-stage CRC and is not recommended as a standalone screening tool. FIT offers a noninvasive alternative with demonstrated population-level utility but is influenced by tumor bleeding patterns and has reduced sensitivity for early or nonbleeding lesions.

The plasma metabolite panel described here captures metabolic alterations independent of tumor bleeding and is fundamentally different from tumor-derived nucleic acid-based assays. The high sensitivity observed in this study, including in early-stage CRC, suggests that metabolomics-based signatures may provide complementary biological information to existing tests. However, the present study was not designed to directly compare performance with FIT, CEA, or other blood-based assays, and such comparisons will require prospective, head-to-head evaluation in appropriately designed cohorts. Accordingly, the five-metabolite panel is best positioned not as a replacement for established screening tools but as a potential adjunctive biomarker that may enhance CRC risk stratification or identify individuals who would benefit from further diagnostic evaluation.

Our study has certain limitations that should be acknowledged. First, although our validation cohort confirmed findings from the discovery cohort, larger and more diverse cohorts are needed to establish the generalizability of these biomarkers across demographic and genetic backgrounds. Second, although our functional assays demonstrated effects on cancer cell migration and invasion, the precise molecular mechanisms by which these metabolites influence CRC progression remain to be investigated. Third, prospective studies are needed to determine whether these metabolites can predict CRC development before clinical manifestation, thereby enhancing their utility for population screening. For example, such a panel could be integrated with existing screening paradigms to prioritize colonoscopic evaluation in individuals with elevated metabolic risk signatures, despite negative fecal testing. Fourth, unmeasured factors, such as smoking, alcohol use, diet, caffeine intake (affecting theophylline levels), and medications, may influence metabolite levels and warrant further investigation. Fifth, with regard to our in vitro functional assays, we acknowledge that the concentration of theophylline used to inhibit migration (450 μg/mL) exceeds standard therapeutic serum concentrations (10–20 μg/mL). While this high concentration was selected to clearly delineate potential phenotypic effects in a short-term assay, the in vivo biological relevance of this specific metabolite likely stems from its role as a biomarker of the systemic metabolic state (e.g., CYP1A2 activity or microbiome function) rather than as a direct circulating tumor suppressor at physiological levels. Future studies should focus on the upstream metabolic pathways regulating theophylline availability. The final limitation of this study is the age imbalance between CRC patients and healthy controls in the discovery cohort. Although multivariate adjustment and subgroup analyses indicated that four metabolites remained independent predictors of CRC, future studies using age-matched cohorts will be necessary to fully eliminate potential confounding effects.

Age is a well-established determinant of systemic metabolic profiles and poses an inherent challenge in plasma-based biomarker discovery for colorectal cancer, particularly given the higher incidence of CRC in older populations. We recognize that the age discrepancy between our cohorts, a common challenge in case-control studies where healthy volunteers are typically younger than cancer patients, poses a risk of confounding. However, several lines of evidence support the disease specificity of our panel. First, multivariate adjustment confirmed that the discriminatory power of 2-piperidone, theophylline, dl-norleucine, and linolenic acid is independent of age. Second, these metabolites were significantly downregulated, even in early-stage (Stages I–II) disease. Since early-stage patients typically lack the sarcopenia or profound metabolic frailty associated with advanced aging or late-stage cachexia, this reduction is unlikely to be a byproduct of senescence. Finally, three of the panel members (2-piperidone, dl-norleucine, and linolenic acid) are established products of the gut microbiome. Their depletion aligns with the known dysbiosis of the CRC microbiome–metabolome axis, providing a distinct biological mechanism that separates these biomarkers from simple markers of aging.

In this study, CRC patients were older than healthy controls, reflecting real-world disease epidemiology rather than an age-matched experimental design. While this imbalance introduces potential confounding effects, several observations support the disease relevance of the identified metabolites. First, four of the five metabolites showed consistent reductions in CRC patients across age strata, with no significant age-dependent variation observed in stratified analyses. Second, these metabolites remained significantly altered in early-stage CRC, where age-related metabolic decline alone would be insufficient to explain the observed differences. Third, integrating multiple metabolites with distinct biochemical origins reduces the reliance on any single age-sensitive marker. Nevertheless, we acknowledge that future studies incorporating age-matched cohorts and multivariable modeling will be essential to fully disentangle age from disease-specific metabolic effects. Accordingly, the current findings should be interpreted as defining a CRC-associated metabolic signature rather than an age-independent screening test.

## 4 Conclusion

This study presents a novel multiplatform metabolomics workflow enhanced by machine learning for colorectal cancer biomarker discovery. By combining untargeted and targeted metabolomics approaches with random forest algorithm-based feature selection, we identified a panel of five metabolites, *N*-methylcytisine, 2-piperidone, theophylline, norleucine, and linolenic acid, that consistently showed decreased concentrations in CRC patients compared with healthy individuals across both discovery and validation cohorts. In addition, the multimarker panel integrating these five metabolites demonstrated a robust diagnostic performance with high accuracy, sensitivity, and specificity, outperforming individual metabolites alone. This panel shows particular promise for early-stage CRC detection, addressing a critical clinical need for noninvasive screening alternatives to colonoscopy.

Phenotypic validation using cell-based assays revealed that these metabolites inhibited the migration and invasion of CRC cell lines, suggesting that, while they act as robust diagnostic biomarkers, they likely reflect systemic host–microbiome interactions and altered hepatic clearance associated with CRC, rather than functioning exclusively as tumor-intrinsic drivers. The identification of three bacterial-derived metabolites (2-piperidone, norleucine, and linolenic acid) among our panel highlights the critical intersection between gut microbiome dysregulation and CRC pathogenesis. This microbiome–metabolome axis represents an essential dimension of CRC biology that warrants further investigation for both biomarker development and therapeutic targeting. In addition to their diagnostic relevance, the identified metabolites demonstrated measurable biological activity in cancer cell phenotypic assays, supporting their relevance to CRC-associated metabolic states. These findings suggest that the five-metabolite panel reflects systemic metabolic environments linked to tumor behavior rather than serving as direct mechanistic drivers of cancer progression.

Our analytical approach demonstrates the value of integrating advanced analytical techniques with computational methods for biomarker discovery. The combination of high-resolution mass spectrometry, machine learning-based feature selection, and functional validation provides a comprehensive framework that could be applied to biomarker research across various cancer types and other diseases. Future research should focus on validating these biomarkers in larger, more diverse patient populations, assessing their performance in prospective screening settings, and exploring the relationship among gut microbiota composition, metabolite production, and CRC development. In this context, the five-metabolite panel may be most appropriately positioned as a risk stratification or adjunctive screening tool, particularly for identifying individuals who warrant further diagnostic evaluation. These efforts could yield insights into both improved early detection methods and potential preventive strategies for colorectal cancer.

In summary, this study identifies a five-metabolite plasma signature that distinguishes CRC patients from healthy individuals and demonstrates robust performance across disease stages. Rather than serving as a standalone diagnostic test, this host–microbiome composite signature reflects systemic metabolic alterations associated with CRC. It may complement existing screening modalities, such as FIT and serum biomarkers. By providing biologically distinct information that is independent of tumor bleeding or tumor-derived nucleic acids, plasma metabolomics may contribute to improved CRC risk stratification and early detection strategies. Future prospective studies directly comparing metabolite-based panels with established screening tools will be essential to define their optimal clinical role.

## 5 Materials and Methods

### 5.1 Sample Collection

Plasma samples were collected from two independent cohorts. The discovery cohort comprised CRC patients (*N* = 172) and healthy volunteers (*N* = 115) from the Human Bioinformation Bank of National Cheng Kung University Hospital (NCKUH). The validation cohort included CRC patients (*N* = 47) from the Bioinformatics Bank of the Medical Research Department of E-Da Hospital and healthy volunteers (*N* = 47) from NCKUH. All participants provided written informed consent, and the study protocols were approved by the Institutional Review Boards of NCKUH and E-Da Hospital (Approval No. B-ER-113-154). Exclusion criteria included prior history of cancer, active infections, metabolic disorders (e.g., diabetes), or recent antibiotic use (<4 weeks before sampling).

Blood samples were collected in ethylenediaminetetraacetic acid (EDTA) tubes and centrifuged at 1600 × *g* for 10 min at 4 °C to isolate plasma. The plasma fraction was aliquoted into polypropylene cryovials (500 μL per aliquot) and stored at −80 °C until analysis, without interim freeze–thaw cycles. A pooled reference plasma sample was prepared for the quality control. Comprehensive clinical data, including age, gender, cancer stage according to the TNM classification system, and primary tumor site, were systematically recorded for all CRC patients enrolled in the study.

### 5.2 LC–MS/MS Analysis

Sample preparation for metabolomic analysis was performed using a standardized protocol.^52^ Briefly, 100 μL of plasma was thawed on ice and spiked with 30 μL of an internal standard (IS) mixture containing l-phenylalanine-3,3-d_2_ (30 nmol/mL; Sigma-Aldrich, St. Louis, MO, USA), cholic-2,2,4,4-d_4_ acid (2 nmol/mL; Sigma-Aldrich), and lysophosphatidylcholine 19:0 (lyso PC19:0, 2 nmol/mL; Sigma-Aldrich) to facilitate normalization and quantification. Protein precipitation and metabolite extraction were performed by adding 520 μL of HPLC-grade methanol, followed by vigorous vortexing (3 min), incubation on ice (10 min), and high-speed centrifugation (15,000 × *g*, 10 min, 4 °C). A 450 μL aliquot of the supernatant was transferred to a fresh microcentrifuge tube and concentrated to dryness using a vacuum concentrator (SpeedVac, Thermo Scientific) without applied heat. The dried residue was reconstituted in 50 μL of methanol/water (3:1, v/v), vortexed thoroughly (3 min), incubated on ice (10 min), and centrifuged again (15,000 × *g*, 10 min, 4 °C) to remove any insoluble material. The final supernatant (45 μL) was transferred to prelabeled autosampler vials for LC–MS/MS analysis.

Metabolomic profiling was performed using a Vanquish ultrahigh-performance liquid chromatography (UHPLC) system coupled to a Q Exactive Plus Hybrid Quadrupole-Orbitrap Mass Spectrometer (both from Thermo Fisher Scientific, Waltham, MA, USA). Chromatographic separation was achieved on an Acclaim VANQUISH C18 UHPLC column (2.1 × 150 mm, 2.2 μm particle size) maintained at 40 °C. The mobile phases consisted of 0.1% formic acid in water (A) and 0.1% formic acid in acetonitrile (B). The gradient elution program was as follows: 1% B (0–2 min), linear increase from 1% to 20% B (2–5 min), linear increase from 20% to 95% B (5–35 min), isocratic at 95% B (35–36 min), return to initial conditions at 1% B (36–36.1 min), and re-equilibration at 1% B (36.1–45 min). The flow rate was maintained at 0.35 mL/min, and the injection volume was 10 μL.

Mass spectrometric data were acquired in positive heating electrospray ionization mode with the following parameters: spray voltage, 3.5 kV; capillary temperature, 250 °C; sheath gas flow rate, 46 arbitrary units; auxiliary gas flow rate, 11 arbitrary units; and spray gas flow rate, 2 arbitrary units. Full MS scans were acquired in the *m*/*z* range of 80–1200 at a resolution of 70,000 (at *m*/*z* 200) with an automatic gain control (AGC) target of 3 × 10^6^ and a maximum injection time of 100 ms. Data-dependent MS^2^ (dd-MS^2^) spectra were acquired for the top 10 most intense precursor ions from each full MS scan using higher-energy collisional dissociation (HCD) with stepped normalized collision energies (NCEs) of 30, 40, and 50. MS^2^ spectra were acquired at a resolution of 17,500 with an AGC target of 1 × 10^5^, a maximum injection time of 50 ms, and an isolation window of 1.5 *m*/*z*. Dynamic exclusion was set to 10 s to minimize repeated fragmentation of the same precursors. All data were acquired in profile mode using Xcalibur software (version 4.1, Thermo Fisher Scientific).

### 5.3 MS Data Processing

Raw mass spectrometric data were processed using MS-DIAL software (version 4.80, RIKEN Center for Sustainable Resource Science, Japan), an open-source program designed for untargeted metabolomics. Data processing included peak detection, peak alignment, deconvolution, and compound annotation. Initial peak extraction parameters were optimized with a minimum peak height threshold set at 30,000, and retention time alignment was performed using the internal standards as anchors. The MS1 mass signals were used for feature selection and subsequent machine-learning analysis. Metabolite annotation was conducted by matching both the MS1 accurate mass and the MS2 fragmentation patterns to the MassBank spectral repository.^53^ Stringent matching criteria were applied with mass tolerance thresholds of 0.01 and 0.025 Da for MS1 and MS2 spectra, respectively. Only annotations achieving a minimum identification score of 80% were retained for subsequent analyses. Peak intensity normalization was performed using the internal standards to correct for technical variations in extraction efficiency and ionization. Analytical reproducibility was confirmed using triplicate injections and spiked recovery experiments (CV < 15%). No data transformations were applied before machine learning analysis to preserve the original scale of metabolite concentrations.

### 5.4 Machine Learning-Based Feature Prioritization and Model Construction

To identify metabolite features that robustly discriminate colorectal cancer (CRC) patients from healthy controls while minimizing overfitting in a high-dimensional data set, we implemented a two-stage machine learning framework that separates feature prioritization from classifier construction.

First, Random Forest (RF) analysis was used exclusively for feature ranking and not as a final predictive model. RF was selected for its robustness to multicollinearity, nonlinear feature interactions, and noise commonly encountered in untargeted metabolomics data sets. The initial data set comprised 146,880 MS1 features extracted from LC–MS profiling. To improve computational tractability and reduce bias associated with single-pass feature selection, the feature space was randomly partitioned into ten nonoverlapping subsets, each analyzed independently with an RF classifier. Within each subgroup, samples were stratified into training (70%) and internal testing (30%) sets. RF models were trained with 20 decision trees, chosen empirically to yield stable feature-importance rankings while avoiding unnecessary model complexity (Figure S1). For each subset, features were ranked by the mean decrease in Gini impurity. Features consistently ranked as informative across subsets were aggregated, yielding a reduced feature set of 1007 candidate discriminatory features. Importantly, RF-derived features were not used directly as a diagnostic classifier. Instead, downstream analyses focused on biological interpretability and clinical relevance. Candidate features were further filtered using receiver operating characteristic (ROC) analysis, with features exhibiting an area under the curve (AUC) > 0.90 prioritized for metabolite annotation. Only metabolites with high-confidence MS/MS spectral matches were retained for further evaluation.

In the second stage, a logistic regression model was built by using quantified concentrations of the selected metabolites. Logistic regression was chosen as the final classifier for its transparency, interpretability, and suitability for clinical translation. Model coefficients were derived from the discovery cohort and applied to the independent validation cohort without retraining. Model performance was assessed using standard metrics, including AUC, sensitivity, specificity, and accuracy, with 95% confidence intervals.

This two-stage framework, RF for robust feature prioritization followed by logistic regression for interpretable model construction, was designed to reduce overfitting while preserving biological insight and clinical applicability.

### 5.5 Assessment of Age and Demographic Effects

To address potential confounding from age and gender imbalances between colorectal cancer (CRC) patients and healthy controls (Table 1), we evaluated the robustness of candidate metabolite associations using complementary approaches, including multivariate logistic regression incorporating age and gender as covariates between metabolite concentrations and age within healthy controls, and stratified subgroup analyses by age, sex, and tumor location using nonparametric statistical tests, with significance defined as *p* < 0.05.

### 5.6 Cell Proliferation Assay

To assess whether the identified CRC-associated metabolites exhibit biological activity relevant to cancer cell behavior, we performed a series of in vitro phenotypic assays focused on cell proliferation and motility.

Cell viability and proliferation were assessed using the Cell Counting Kit-8 (CCK-8; Enzo Life Sciences, New York, USA) according to the manufacturer’s instructions. Human colorectal cancer cell lines HCT116 and T5088 were cultured in McCoy’s 5 A medium (Gibco Life Technologies, USA) and DMEM/F-12 medium (Invitrogen, USA), respectively. Both media were supplemented with 10% fetal bovine serum (FBS, GIBCO, USA) and 1% penicillin–streptomycin (GIBCO, USA). Cells were maintained at 37 °C in a humidified atmosphere containing 5% CO_2_. Cells were seeded in 96-well flat-bottomed microplates at a density of 5 × 10^3^ cells per well and allowed to adhere overnight. The candidate metabolites (*N*-methylcytisine, 2-piperidone, theophylline, dl-norleucine, and linolenic acid) were dissolved in appropriate vehicles and diluted in a serum-free medium to achieve final concentrations ranging from 0.1 to 100 μM. Treatment solutions were prepared fresh before each experiment. After 24 h of treatment, 10 μL of the CCK-8 solution was added to each well and incubated for an additional 2 h at 37 °C. Absorbance was measured at 450 nm using a microplate spectrophotometer (U2800A, Hitachi, Tokyo, Japan). Cell proliferation was calculated as a percentage relative to vehicle-treated controls using the formula: Proliferation (%) = [(Absorbance of treated cells – Absorbance of blank)/(Absorbance of control cells – Absorbance of blank)] × 100. All experiments were performed in technical triplicate and repeated three times independently.

### 5.7 Transwell Cell Migration Assay

Cell migratory capacity was evaluated by using Transwell permeable supports with polycarbonate membranes containing 8 μm pores (Corning, NY, USA). HCT116 and T5088 cells were serum-starved for 12 h prior to the assay to minimize proliferation-dependent effects. Cells were harvested and resuspended in serum-free medium containing the candidate metabolites at physiologically relevant concentrations (*N*-methylcytisine: 8 ng/mL; 2-piperidone: 4 μg/mL; theophylline: 450 μg/mL; dl-norleucine: 220 μg/mL; linolenic acid: 70 μg/mL) and seeded in the upper chamber at a density of 5 × 10^4^ cells in 50 μL of medium per insert. The lower chamber contained 500 μL of complete medium supplemented with 10% FBS to serve as a chemoattractant. After 18 h of incubation at 37 °C in 5% CO_2_, nonmigrated cells remaining on the upper surface of the membrane were carefully removed using a cotton swab. Migrated cells adhering to the underside of the membrane were fixed with 100% methanol for 30 min at room temperature and stained with a 1% crystal violet solution for 1 h. Quantification was performed by imaging five random microscopic fields per insert. Images were analyzed using ImageJ software (NIH, version 1.54a) with automated cell counting. Migration was expressed as the mean number of migrated cells per field ±standard deviation relative to vehicle control. Each experiment was repeated independently three times.

### 5.8 Wound-Healing Assay

The wound healing assay was conducted using culture inserts (ibidi GmbH, Gräfelfing, Germany) placed in 24-well plates to generate uniform, cell-free gaps. HCT116 and T5088 cells were seeded into each compartment of the inset at a density of 5 × 10^5^ cells/mL in the complete growth medium and incubated for 24 h to allow the formation of confluent monolayers. After cell attachment, the insets were carefully removed using sterile forceps to initiate the wound-healing process, resulting in a defined cell-free gap of approximately 500 μm. Cell monolayers were gently washed twice with PBS to remove detached cells, and the medium was replaced with a serum-free medium containing the candidate metabolites at the concentrations described above. Wound closure was monitored by capturing phase-contrast images at 0 and 24 h postwounding using a light microscope. For each condition, three different positions along the wound were imaged and analyzed. The wound area was quantified using the ImageJ software, and the percentage of wound closure was calculated using the formula: Migratory area (%) = [(Area at 0 h – Area at 24 h)/Area at 0 h] × 100. All experiments were performed in triplicate and repeated independently three times.

## Supplementary Material



## Data Availability

The mass spectrometry metabolomics data supporting the findings of this study, including the processed data set containing the identified metabolite features and their quantification across all samples, is available from the corresponding author upon request. Clinical data for the patient cohorts are available in deidentified form from the corresponding author, subject to approval from the relevant Institutional Review Boards of NCKUH and E-Da Hospital. Ethics approval and consent to participate: The study was approved by the Institutional Review Boards of National Cheng Kung University Hospital and E-Da Hospital (Approval No. B-ER-113-154). Written informed consent was obtained from all individual participants included in the study.

## Notes

Additional results (Figure S1–S4) including the enhancement in model performance, ROC curves of annotated metabolites, age-adjusted associations between plasma metabolites and colorectal cancer risk, and age-associated distribution of plasma theophylline concentrations in healthy controls (PDF)
