# Supplementary material for: Machine Learning-Assisted Plasma Metabolomics Identifies a Five-Metabolite Panel for Colorectal Cancer Detection
Source: ACS Omega. 2026 Apr 16;11(16):24635–49. doi: 10.1021/acsomega.6c00857 (PMC13129852; doi:10.1021/acsomega.6c00857)
Supplement: Supplementary file 1 [file ao6c00857_si_001.pdf]

Machine Learning-Assisted Plasma Metabolomics Identifies a Five-Metabolite Panel for Colorectal Cancer Detection

Jun-Kai Wong [a§], Chung-Hsien Lin [a§], Hsin-Yi Wu [b], Yen-Ping Lin [c], Chiau-Jun Chu [c], Pang-Hung Hsu [d,e,f*], Chung-Fa Chang [a,g,h,i*]

[a]Department of Medical Laboratory Science and Biotechnology, College of Medicine, National Cheng Kung University, Tainan 70101, Taiwan

[b]Instrumentation Center, National Taiwan University, Taipei 106, Taiwan

[c]Public Health Bureau, Tainan City Government, Tainan 701, Taiwan

[d]Department of Bioscience and Biotechnology, National Taiwan Ocean University, Keelung 202, Taiwan

[e]Institute of Biochemistry and Molecular Biology, National Yang Ming Chiao Tung University, Taipei 112, Taiwan

[f]Center of Excellence for the Oceans, National Taiwan Ocean University, Keelung 202, Taiwan

[g]Institute of Basic Medical Science, College of Medicine, National Cheng Kung University, Tainan 70101, Taiwan

[h]University Center for Bioscience and Biotechnology, National Cheng Kung University, Tainan 70101, Taiwan

[i]Department of Pathology, National Cheng Kung University Hospital, College of Medicine, National Cheng Kung University, Tainan, 70101, Taiwan

[*] E-mail: phsu@ntou.edu.tw

[*] E-mail: affa@mail.ncku.edu.tw

[§] *J.K.W. and C.H.L. contributed equally to this work*

Supporting Information

To address the substantial volume of untargeted MS/MS data acquired via HRMS and to mitigate the subjectivity inherent in human selection, a random forest algorithm was used for feature selection. Within the feature selection procedure, the primary parameter for optimization is the count of trees, denoted as "n_estimators." While increasing the number of decision trees typically enhances performance, too many can lead to increased computational demands or overfitting. Conversely, too few trees might result in underfitting. Consequently, identifying the optimal number of decision trees is paramount.

In Supplementary Figure 1, experiments were conducted with decision tree quantities up to 200. However, it became evident that once the number of decision trees exceeded 20, the model's performance showed negligible improvement. As a result, this study determined that 20 decision trees represented the optimal choice for this parameter.

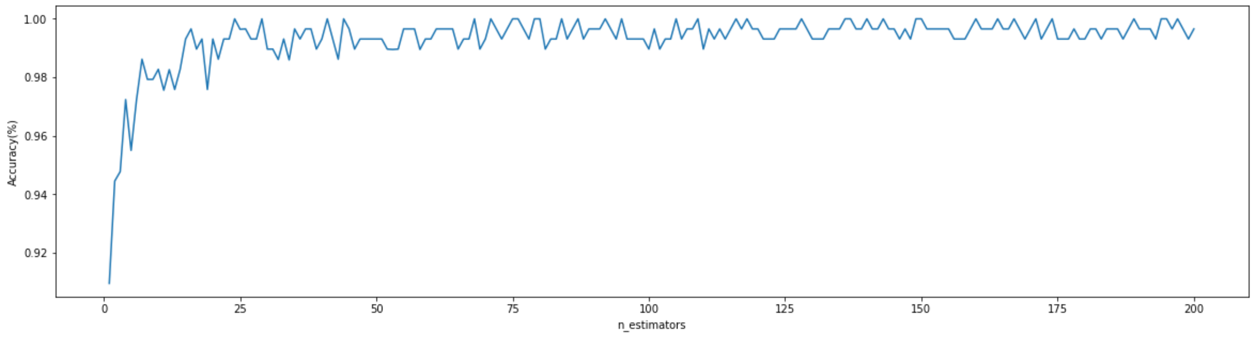

Figure S1: Enhancement in Model Performance with Increasing Decision Tree Count (n_estimators). Notable performance improvements are observed as the number of decision trees increases. However, beyond a threshold of 20, no substantial further performance enhancement is discernible.

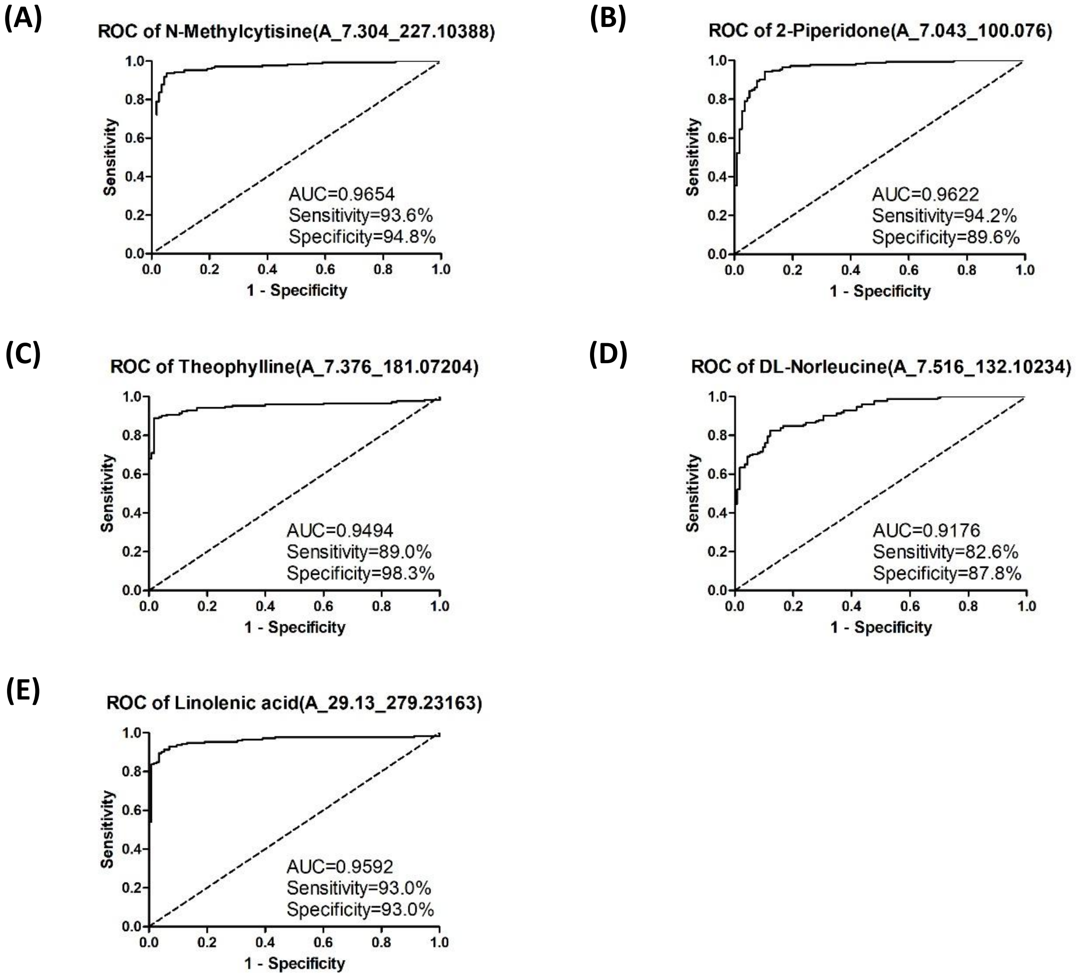

Figure S2. The ROC curves of annotated metabolites between the healthy and CRC groups based on the discovery group. (A) N-Methylcytisine, (B) 2-Piperidone, (C) Theophylline, (D) DL-Norleucine, (E) Linolenic Acid.

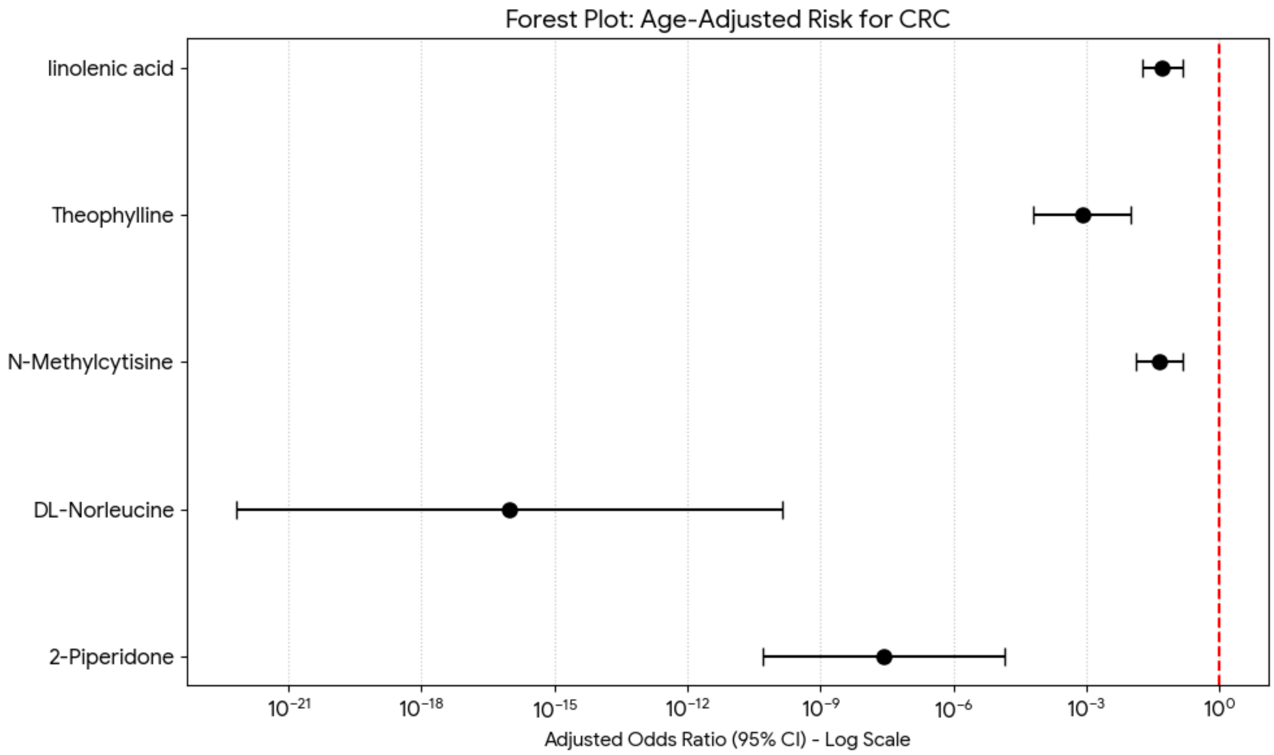

Figure S3. Age-adjusted associations between plasma metabolites and colorectal cancer risk. Forest plot of adjusted odds ratios (ORs) and 95% confidence intervals (CIs) for colorectal cancer (CRC) associated with each metabolite, derived from multivariate logistic regression models adjusting for age. Odds ratios are displayed on a logarithmic scale. The vertical dashed red line indicates the null value (OR = 1). Four metabolites (2-piperidone, DL-norleucine, theophylline, and linolenic acid) remain significantly associated with CRC risk after demographic adjustment, whereas N-methylcytisine shows partial attenuation, consistent with age-stratified analyses.

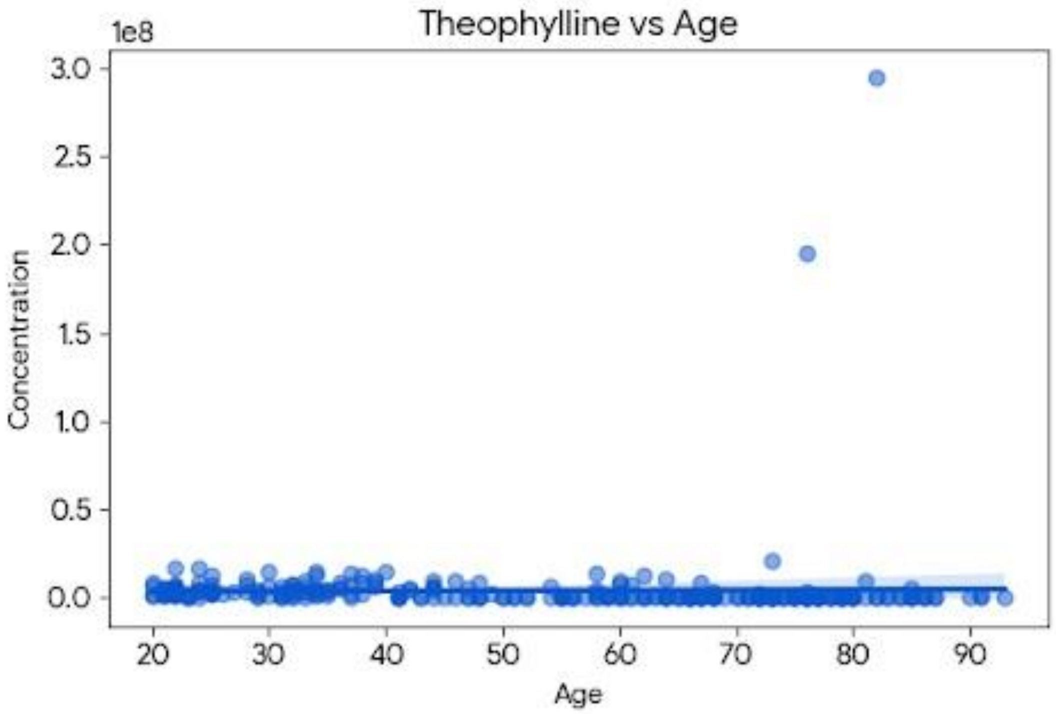

Figure S4. Age-associated distribution of plasma theophylline concentrations in healthy controls. The scatter plot shows the relationship between age and plasma theophylline concentration in healthy control subjects (n = 115). The association was evaluated using Spearman's rank correlation and showed no significant correlation with age ($\rho = 0.02$, p = n.s.). The solid line indicates the fitted trend shown for visualization purposes only.
